# Hospitalized for poverty: orthopaedic discharge delays due to financial hardship in a tertiary hospital in Northern Tanzania

**DOI:** 10.1186/s41256-022-00265-z

**Published:** 2022-09-02

**Authors:** Joy E. Obayemi, Elizabeth B. Card, Octavian Shirima, Ajay Premkumar, Honest Massawe, Neil P. Sheth

**Affiliations:** 1 Perelman School of Medicine, University of Pennsylvania, Philadelphia, USA; 2 Kilimanjaro Christian Medical Centre (KCMC), Moshi, Tanzania; 3 Hospital for Special Surgery, NewYork, USA; 4 Pennsylvania Hospital, Hospital of the University of Pennsylvania, Philadelphia, USA

**Keywords:** Global surgery, Orthopedic injury, Road traffic crash, Health policy, Ethics, Financial hardship, LMIC

## Abstract

**Background:**

Musculoskeletal injury contributes significantly to the burden of disease in Tanzania and other LMICs. For hospitals to cope financially with this burden, they often mandate that patients pay their entire hospital bill before leaving the hospital. This creates a phenomenon of patients who remain hospitalized solely due to financial hardship. This study aims to characterize the impact of this policy on patients and hospital systems in resource-limited settings.

**Methods:**

A mixed-methods study using retrospective medical record review and semi-structured interviews was conducted at a tertiary hospital in Moshi, Tanzania. Information regarding patient demographics, injury type, days spent in the ward after medical clearance for discharge, and hospital invoices were collected and analyzed for orthopaedic patients treated from November 2016 to June 2017.

**Results:**

346 of the 867 orthopaedic patients (39.9%) treated during this time period were found to have spent additional days in the hospital due to their inability to pay their hospital bill. Of these patients, 72 patient charts were analyzed. These 72 patients spent an average of 9 additional days in the hospital due to financial hardship (range: 1–64 days; interquartile range: 2–10.5 days). They spent an average of 112,958 Tanzanian Shillings (TSH) to pay for services received following medical clearance for discharge, representing 12.3% of the average total bill (916,840 TSH). 646 hospital bed-days were spent on these 72 patients when they no longer clinically required hospitalization. 7 (9.7%) patients eloped from the hospital without paying and 24 (33.3%) received financial assistance from the hospital’s social welfare office.

**Conclusions:**

Many patients do not have the financial capacity to pay hospital fees prior to discharge. This reality has added significantly to these patients’ overall financial hardship and has taken hundreds of bed-days from other critically ill patients. This single-institution, cross-sectional study provides a deeper understanding of this phenomenon and highlights the need for changes in the healthcare payment structure in Tanzania and other comparable settings.

## Introduction

As the frequency of road traffic crashes (RTCs) rises in Sub-Saharan Africa, the need for effective and timely orthopaedic care has become increasingly important. Musculoskeletal injuries can cause significant disability and preclude working-age men and women from returning to the work force [1, 2]. Due to limited material resources and surgical capacity, many patients with musculoskeletal injuries are subject to long treatment regimens that involve weeks of skeletal traction [3]. The increased frequency of RTCs and the potential for long length of stay (LOS) contributes to the burden orthopaedic injuries place on health care centers in low to middle income countries (LMIC).

This burden can be further exacerbated by a patient’s financial status. In rural Tanzania, surgical treatment requires up-front payment, which is cost prohibitive for most of the population. Additionally, patients are not permitted to leave the hospital until their hospital bill has been paid, a policy also implemented in many surrounding countries [4, 5]. Given the lack of infrastructure in these rural settings, it is often impossible for hospitals to mail a patient the final bill for services or to even contact a patient following discharge. Hospitals therefore find that payment prior to discharge is the only way to replenish operational costs and to continue providing much-needed care in the region. While this policy assures that the hospital is paid for its services, it can be financially crippling for patients when only 15% of Tanzanian citizens are covered by health insurance [6].

The unintended consequence of required payment prior to discharge is that patients, even when medically cleared, are forced to remain in the hospital until their bill has been paid. At Kilimanjaro Christian Medical Center (KCMC), a tertiary hospital in Moshi, Tanzania, patients in this situation have been informally labeled by the Orthopaedic Department as *D-Still—Discharged* but *Still* on the ward. These patients are responsible for additional costs accrued during this time, further increasing the financial burden. As a result, other patients requiring treatment are being denied hospital admission due to inadequate bed space. Though this phenomenon has been commented on from a human rights perspective, limited research has been published on this topic [5, 7–12]. Using the KCMC Orthopaedic Department as a case-study, this study aims to: (1) determine the prevalence of *D-Still* patients during a specified period, (2) quantify the additional financial burden placed on *D-Still* patients, (3) calculate the bed-days spent on *D-Still* patients who no longer required medical care, and (4) understand the complexities of social welfare assistance in this setting.

## Methods

### Research design and setting

At KCMC, daily nursing status notes for each orthopaedic ward patient between November 2016 and June 2017 were accessed from the *Ward Round Book* (WRB). The name and hospital ID of every *D-Still* patient as well as the total number of patients in the Orthopaedic Department over the study period were documented. This information was used to request patient records from the medical records office. This retrospective research was performed in June of 2017, during which time lead authors JEO and EBC remained at KCMC and were fully immersed in the work of the Orthopaedic Department, attending morning clinical meetings and rounding on patients.

While on site, key informants were identified to provide additional, nuanced information about this phenomenon via individual interviews. After detailed discussions with clinical providers on the ward and weeks of observation, a purposive sampling method was employed to identify these individuals as the most suited to serve as key informants and to provide detailed and holistic information regarding the discharge process [13].

### Interview development

Interview questions were developed collectively with input from all authors. A detailed review of the final questions was performed by OS and other Tanzanian authors to assure comprehensibility given the cross-cultural nature of this work. Both open-ended and job-specific questions were asked to give the informants the opportunity to describe the discharge process in detail as it pertains to patients with financial difficulty (See Table 1). The goal of these interviews was to gain a deeper understanding of the discharge protocol for patients in the department and the process of having a hospital bill covered by the social welfare office.

**Table 1:** Semi-structured interview question guide

1. Can you describe the process of discharging a patient? What steps are involved?
2. What are some reasons that there might be a delay in discharging a patient?
3. Who exactly collects the final payment a patient makes?
4. How do you confirm that a patient has paid before processing the discharge?
5. How does nursing staff work with the social welfare office to discharge a patient?
6. Do different nurses follow different discharge procedures?
7. Typically, at what time are patients formally discharged?
8. Is there a difference between the discharge process for those discharged during the day versus at night?
9. What happens during ward rounds to a patient that has been discharged but is still in the ward?
10. How does treatment of a patient change when he is cleared for discharge but still remains in the ward? Is the patient checked on as often? Does care continue?
11. How does the social welfare office identify patients in need of financial support?
12. What is the process of evaluating patients for financial assistance?
13. How do you confirm that the patient has financial need?
14. How does the social welfare office collaborate with providers on the ward caring directly for patients?
15. What are some key aspects of the discharge and financial assistance process that are important for us to understand as non-Tanzanian providers?

### Data collection

**Fig. 1:**
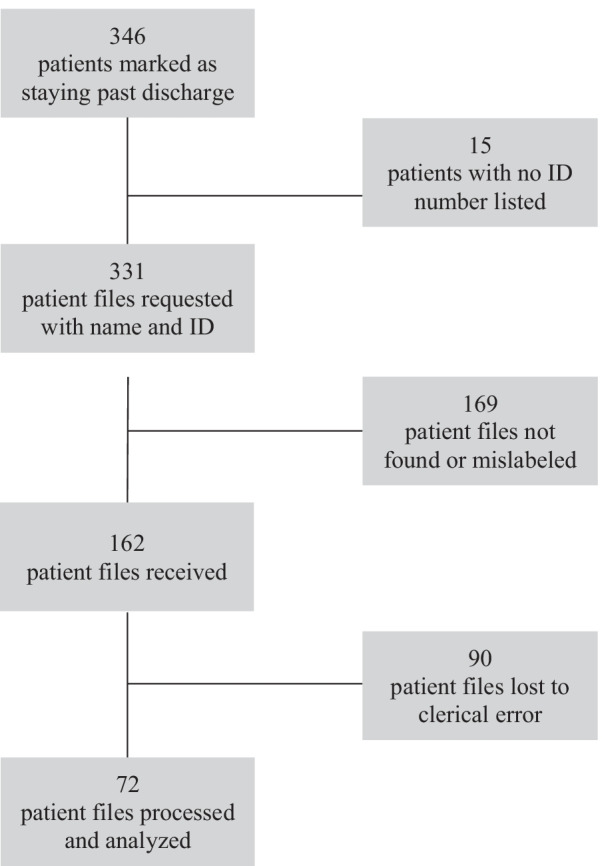
Flow of medical records requested and processed

Due to limitations inherent to a paper medical record system, a final cohort of 72 medical *D-Still* records was gathered and information regarding the following variables was collected: sex, age, injury type, and treatment regimen. Descriptive statistics surrounding the overall bill paid, social welfare assistance, and length of stay were also computed (Fig. 1).

Semi-structured interviews in English were conducted with the lead social welfare officer at KCMC and the head nurse of the orthopaedic ward by two authors (JEO and EBC). Both participants were fluent in English and gave their informed consent to be interviewed. One, 30-min interview was performed with each participant individually. At the time of the interviews, JEO and EBC were conducting this research as medical students from the United States and JEO had graduate level training in ethnography and qualitative social science research. Audio from the interviews was not formally recorded due to participant preference, but extensive notes were taken during the interviews and later analyzed.

### Data processing and analysis

For the quantitative data, descriptive analytics was performed and statistical significance was determined using bivariable logistic regression to explore associations between variables of interest. A *p*-value < 0.05 was used to delineate significance. This analysis was performed with R studio software (Version 1.2.5042).

For the qualitative data, field notes from the interviews were transcribed and coded thematically by JO and EC. The purpose of the interviews was to gain a deeper understanding of the *D-Still* phenomenon from the perspective of clinicians and hospital staff. Descriptive analytics and thematic analysis were performed to identify the workflow in the wards when managing *D-Still* patients. Using this qualitative, low-inference, descriptive approach, a “comprehensive summary of an event” as Sandelowski et al. describe was developed [14, 15]. The interviews were grounded in naturalistic inquiry in an attempt to fully characterize how these patients are treated. Following the purposive sampling of key informants, two iterations of coding were used to glean meaningful themes from the interview data. Themes were discussed with the entire authorship team, though feedback from interview participants was unable to be solicited. The qualitative component of the research presented here is in accordance with the COREQ (Consolidated Criteria for Reporting Qualitative Research) checklist [16]. No additional software was needed for this qualitative analysis.

### Ethics statement

This study received local institutional review board approval at Kilimanjaro Christian Medical College (Research Ethical Clearance No 2220).

## Results

### Medical record review

Over the study period, a total of 867 patients were treated as inpatients, of which 346 (39.9%) were classified as *D-Still* according to the WRB.

Of the 72 patients in the final cohort, the average age was 34 years-old (range 1–87) and 57 (79.2%) were males (Table 2). Patients predominantly sustained a lower extremity injury (70, 74% of total injuries). Most injuries were closed fractures with open fractures as the second most common injury type. Most patients (59, 84%) underwent at least one surgical procedure. The average LOS for treatment in the Orthopaedic Department was 36 days, with a median of 16.5 days and range of 0 to 381 days. Seven patients (9.7%) absconded from the hospital without paying their medical bills (Table 3).

**Table 2:** Patient demographic information

Variables	n	Percentage (%)
*Age (Median* = *25.5)*		
1–20	25	35
21–44	24	33
44 +	23	32
*Sex*		
Female	15	21
Male	57	79
*Injury location(s)*		
Femur	38	53
Tibia and/or fibula	24	33
Other lower extremity	8	11
Upper extremity	12	17
Head	8	11
Pelvis	4	6
*Injury type*		
Closed fracture	41	57
Open fracture	33	46
Osteomyelitis	2	3
Other injury type	3	4
*Treatment*		
Traction	11	15
1 Surgery	45	63
2 Surgeries	14	19
N/A (None documented)	2	3
Total	72	100

**Table 3:** Patient hospitalization information

Variables	n	Percentage (%)
*Length of stay (before medical clearance for discharge)*		
≤ 7 days	23	32
7–30 days	27	37.5
> 30 days	22	30.5
*Social welfare recipient*		
Yes	24	33
No	48	67
*Absconded*		
Yes	7	9.7
No	65	90.3
Total	72	100

The 72 *D-Still* patients spent an average of 9 (median of 4 days, interquartile range 2–10.5 days) extra days as an inpatient after meeting medical criteria for discharge (Table 4). This equated to an average of 112,958 TSH (equivalent to $48.81 USD as per foreign exchange rate on May 26, 2020) additional cost, which represented on average 12.3% of the complete hospital bill. The range of payments made for this post-discharge period was 0 TSH—1,960,000 TSH ($846.94 USD). Of all *D-Still* patients analyzed, a total of 646 hospital bed-days were used by these patients without medical necessity. There was a general trend of patients with osteomyelitis staying longer in the hospital than patients with other injury types (*p* = 0.08).

**Table 4:** Hospital bills and bed-days spent on D-Still patients (n = 72)

Description of data	Mean	Minimum	Maximum	Standard deviation
Extra days spent in hospital*	9	1	64	13
Total hospital bill	916,840 TSH	174,600 TSH	9,688,750 TSH	1,156,080 TSH
Total payment for Post-discharge period**	112,958 TSH	0***	1,960,000 TSH	254,442 TSH

*Represents the number of days spent in the hospital by *D-Still* patients following medical clearance for discharge

**These payments include coverage of hospital bed and food fees, medications administered daily, changing of dressings, and other elements of patient care allotted to all patients staying in the ward

***Patients who paid 0 TSH for this period were those that absconded from the ward without making any payments

Information was also collected from the medical records regarding patients who received assistance from KCMC’s social welfare office. 24 (33.3%) patients received documented financial assistance (Table 3). These patients stayed for an average 17 days (range 0–64) after they had been medically cleared, compared to the overall average of 9 days. Furthermore, patients that received social welfare assistance exhibited a significantly longer LOS following medical clearance for discharge than patients that did not receive any assistance (*p* = 0.0002). Even patients who only received partial social welfare assistance had a significantly longer LOS than those who paid independently (*p* = 0.006).

### Interview findings

Additional information regarding the discharge protocol and the management of patients with financial hardship was gathered through semi-structured interviews with key informants on the ward. There were three major themes discussed in these interviews: (1) Discharge process, (2) Reasons for delay of discharge, and (3) Identifying financial distress. The first interview was conducted with the head nurse of the Orthopaedic Department. In this interview, she spoke primarily about the details of the discharge process. She described that the process of discharging a patient began with the doctor’s determination that the patient was clinically stable and no longer required inpatient care. Following this, a form detailing all of the care that the patient had received during their stay was sent to the hospital billing room. This form was an invoice for medications, procedures, hospital bed-days, and other aspects of patient care. Relatives of the patient were then requested to pay this bill at the billing office, after which the patient was given a discharge summary and a return date for outpatient follow-up. When patients had a LOS of more than 2–3 days after discharge, the head nurse sent a note to the social welfare office to make them aware of the situation. Discharges always occurred at the same time every day, and the same protocol was followed by each nurse under the direction of the head nurse.

Regarding reasons for delay of discharge, the head nurse reported that the main reason for delay was financial difficulty experienced by the patient. Other reasons for delay included (1) the need for a final x-ray to confirm injury status and (2) the application of Plaster of Paris (POP). Due to limitations in manpower for transportation services and machine availability, only 5 or 6 patients could have an x-ray performed each day. POP application also required materials and personnel that were not always readily available. The head nurse specifically commented that *D-Still* patients “do not stay longer than 2–3 weeks” and that “the social welfare always helps.”

In the second interview with a social welfare officer, more was learned about the third theme: identifying financial distress. She revealed that patients in financial distress were either (1) identified by personnel in the different departments of the hospital or (2) identified by a team of 6 individuals who travel around the hospital to check for patients that could benefit from social welfare assistance. Signs that a patient could need financial assistance included the absence of visitors, a lack of food provision by loved ones, and the presence of tattered clothing. The welfare office went as far as calling relatives and visiting patients’ homes to speak with village leaders and neighbors to confirm the need for financial assistance. When asked how they evaluate if a patient is poor enough to warrant social welfare, the officer replied, “You know when someone is poor. You see how they live, if they don’t have a house or food to eat.”

After making this assessment of the patient’s economic standing, the social welfare office determined if they would cover the entirety or a portion of the payment for hospital services. At times they would grant bonds to patients, asking patients to pay a small sum immediately and make additional payments upon returning to clinic. Patients who owed money often never paid and never returned to clinic to pay the money they owed. Per the social welfare officer, some patients even changed their name upon returning for additional care to avoid making payments.

The social welfare office provided general statistics about the number of patients they assisted and the amount of money that was forgiven on a regular basis. In a typical month, the office will help about 30 patients on the inpatient ward who need financial assistance. Over 250 patients receive assistance in a typical year, with the office spending 14,000,000TSH or more per month to assist patients**.** She also reported that each year > 200 patients on average abscond from the hospital without making their payments, which results in net loss for the hospital. During the interview, it was emphasized that the hospital would not let patients die due to an inability to pay. This was further supported by the communal and family-oriented nature of the welfare office and their approach to working with patients and families, highlighted in the following quote:

“We don’t look at how much money it will cost. If you are poor or you are rich, you need help. We don’t look at money. You only have one life to live. We are not here to see people die.”— Social Welfare Officer

## Discussion

The data presented here characterizes the financial hardship posed by inpatient orthopaedic care in rural Tanzania and highlights the hospital resources spent on patients with financial hardship. In settings like Northern Tanzania, hospital resources are often inadequate to care for those in need of medical care. At KCMC, this study has shown that many hospital beds and hundreds of hospital bed-days are in fact taken by patients who simply cannot afford to leave. Approximately 40% of patients receiving orthopaedic care spent additional time in the ward due to their inability to pay promptly for services. The 72 patients further analyzed in this study stayed an average of 9 extra days in the hospital receiving food, dressing changes, and medical services that could be managed in the outpatient setting. This also delays the rehabilitation and physical therapy that occur outside of the hospital that can help to improve patient functionality and mobility following serious musculoskeletal injury [17]. Spending bed-days on patients in financial distress limits the access other patients have to emergent care. It also limits the supplies, such as wound dressings and medications, that are available for other patients in need [18, 19].

Patients are taking additional time to pay their discharge invoices because they often do not have the means to immediately cover the payment [5]. This problem is then compounded by the fact that each additional day the patient spends in the hospital will increase the total amount that the patient is required to pay, with no decrease in the daily hospital-bed fee. As reported above, on average 13% of the bill that patients pay is to cover expenses accrued after necessary inpatient care was completed. This represents a significant financial burden. Patients in this study were asked to pay an average of 916,840 TSH in total charges to the hospital. We know that the median per capita monthly income in the Kilimanjaro region of Tanzania is 8,732 TSH and 16,472 TSH in Dar es Salaam [20]**.** Therefore, even for wealthier individuals living in Dar es Salaam, the average total hospital charge for these patients—916,840 TSH—represents 464% of the median annual income.

Several studies in the literature have documented the financial distress that patients in LMICs can experience when paying for healthcare [21–23]. One study found that 81.1% of patients faced catastrophic expenditures when managing severe malaria in children. In this case, catastrophic expenditures were defined as spending at least 40% of the household’s income on medical bills [24, 25]**.** In a study of orthopaedic care in Northern Tanzania specifically, 73.7% of patients with musculoskeletal injuries reported that their healthcare costs were a catastrophic burden with 75% of patients reporting that their healthcare payments exceeded their monthly income [26]. Over 40% of these patients also reported that their injuries led to unemployment following hospitalization, further emphasizing the length and pervasive nature of the financial hardship caused by healthcare treatment and costs [26]. Reducing the financial toxicity of orthopaedic care or restructuring the current payment model could allow patients to leave the hospital in a timely fashion with less impact on their livelihoods while simultaneously making more inpatient beds available for new patients to receive medical care.

Devakumar et al. referred to the phenomenon explored in this study as the taking of “medical hostages” by hospitals in resource-poor settings [7, 8, 27]. Decreasing the amount of time that patients are subjected to detainment may improve their overall experience of healthcare, their mental health, and the likelihood that they will return to the hospital for future care. Patients who abscond from the hospital are certainly a source of frustration for hospital administrators, but one can imagine how the stress and hopelessness associated with this financial predicament could lead patients to physically escape from hospital care.

As reported above, receiving social welfare was associated with significantly longer stays in the orthopaedic department. This is perhaps explained by the fact that patients must be in the hospital for several days following medical clearance before the social welfare office becomes aware of their case. At that point, social welfare officers investigate whether or not the patient is truly in financial distress, as the officer described in her interview. This can involve calls to relatives and even visits to the individual’s home, which may be miles away from the hospital. This process occurs while the patient is still accumulating charges as a patient in the ward. The length of the social welfare review process may also account for the fact that patients receiving financial assistance have a much longer stay than those who perhaps relied on their social networks to raise the appropriate funds. As a result, one must wonder if social welfare services intervene too late, often acting as a last resort for frustrated patients and hospital staff. Engaging with patients who are concerned about finances at the beginning of their care may be a way to increase the impact of this service. Additionally, the process of determining if a patient has the means to pay for care, as described by the social welfare officer, appears to be subjective and lacking specific calculations or consistency. Unlike what the head nurse indicated in her interview, only 33% of the patients in this study received social welfare and the evidence suggests that perhaps many more of them would have benefited from some assistance had they been formally evaluated.

The authors hope that the work presented here will inform future health policy changes in Tanzania as well as other LMICs with similar payment structures. Given the toll that the “payment-before-discharge” financial model can have on a patient’s financial health and on hospital resources, alternative payment models should be fully explored. Early identification of patients in financial distress may also help to mitigate the far-reaching impact of a prolonged hospitalization.

There were limitations inherent to this study. The number of accessible patient records was severely limited by the lack of detailed information provided in the WRBs, the mislabeling and misplacement of files, and clerical errors in the medical records office. These challenges highlight the difficulty of working with paper records in low resource settings. It has been established in the literature that retrospective chart reviews are limited by proper documentation and handling of charts [1]. In addition, there was limited access to detailed demographic information for each patient, which could have revealed any unexpected bias in this *D-Still* population. Due to a lack of a clear reference population, it is unknown if the *D-Still* patients described here are truly representative of the patients treated in the orthopaedic department or at KCMC in general. It is reassuring, however, that the gender and injury distribution reported in this population corresponds to that reported in prior studies in this setting [28].

There are also inherent limitations to the qualitative aspect of this study. Thematic saturation was not feasible from the two interviews performed with the key informants. Therefore, the study results report primarily descriptions of the phenomenon rather than a comprehensive theory. This purposive sampling was chosen after extensive discussion with Tanzanian providers on the ward and Tanzanian co-authors, who felt that it would be the best way to learn more about the discharge process while being mindful of the limited availability of these providers on the ward. However, interviews with additional nursing and social work staff would have helped to elucidate additional nuance in the discharge process for *D-Still* patients. The interview subjects were also unable to be contacted following thematic analysis, which would have helped to evaluate the accuracy of the analysis in a co-constructivist manner [29]. As is always the case with research performed by individuals from another culture and from another native language, cultural differences may have impacted the communication between interview subjects and the interviewers [30]. However, collaboration and review of the data with Tanzanian providers and co-authors provides some reassurance that there were not gross contextual misinterpretations.

## Conclusions

The data presented here provides a foundation for the future work needed to fully characterize this phenomenon in the hospital setting. This study uses the orthopaedic department of a tertiary hospital in Tanzania as a case-study on the financial burden of surgical care and hospital-bed efficiency in the Sub-Saharan region. Though this work was performed at a single institution, many hospitals in the region have comparable policies and face the same infrastructural barriers to receiving payment for medical treatment [5]. Quantifying the bed-days spent on patients that have completed their medical care provides the specificity needed to fully appreciate this problem and to formulate potential solutions. The evidence presented here also allows for a clear appreciation of the devastating financial consequences for patients with musculoskeletal injuries. This work has the potential to serve as a launching point for improvements in hospital efficiency in LMICs and for attempts to restructure patient payment models.

## Data Availability

Data sharing not applicable to this article as no generalizable datasets were generated or analyzed during the current study. Please contact author for any specific data requests.

## Abbreviations

LMIC: Low to middle income country
KCMC: Kilimanjaro Christian Medical Center
D-Still: Patients who are clinically ready to be discharged but who remain in the hospital due to financial hardship
WRB: Ward round book
LOS: Length of stay
POP: Plaster of Paris
RTC: Road traffic crash
USD: United States dollar
TSH: Tanzanian shilling
